# Preparing for the Next Pandemic: An Asian National Cancer Centers Alliance (ANCCA) Initiative

**DOI:** 10.31557/APJCP.2021.22.9.2945

**Published:** 2021-09

**Authors:** Laureline Gatellier, Sok King Ong, Tomohiro Matsuda, Noraslinah Ramlee, Fen Nee Lau, Suhana Yusak, H.R. Soeko Werdi Nindito D, Achmad Mulawarman Jayusman, Ayu Hutami Syarif, Kishore K. Pradhananga, Aasim Yusuf, Giang Nguyen Huong, Manju Sengar, CS Pramesh, Alireza Mosavi Jarrahi, Tatsuya Suzuki, William Y Hwang

**Affiliations:** 1 *National Cancer Center, Japan, 5-1-1 Tsukiji, Chuo-ku, Tokyo, Japan. *; 2 *Early Detection and Cancer Prevention Services, The Brunei Cancer Centre, Pantai Jerudong Specialist Centre, Brunei Darussalam. *; 3 *National Cancer Institute, 4, Jalan P7, Presint 7, 62250 Putrajaya, Wilayah Persekutuan Putrajaya, Malaysia. *; 4 *Dharmais Hospital - National Cancer Center, Jalan Letjend S. Parman No.84-86 Kecamatan Palmerah, Kota Jakarta Barat, DKI Jakarta, Indonesia. *; 5 *Kathmandu Cancer Center, Tathali, Bhaktapur, Nepal. *; 6 *Shaukat Khanum Memorial Cancer Hospital and Research Centre, Lahore, Pakistan. *; 7 *National Cancer Institute, National Cancer Hospital, Hanoi, Vietnam.*; 8 *Tata Memorial Centre, Homi Bhabha National Institute, Dr Ernest Borges Rd, Parel, Mumbai, Maharashtra 2, India. *; 9 *Medical School, Shahid Behshti University of Medical Sciences, Tehran, Iran. *; 10 *National Cancer Centre of Singapore, 11, Hospital Crescent, Singapore. *; 11 *Duke-NUS Medical School, 8 College Road, Singapore. *

**Keywords:** Asia, Coronavirus, cancer center, patients

## Abstract

The COVID-pandemic has shown significant impact on cancer care from early detection, management plan to clinical outcomes of cancer patients. The Asian National Cancer Centres Alliance (ANCCA) has put together the 9 “Ps” as guidelines for cancer programs to better prepare for the next pandemic. The 9 “Ps” are Priority, Protocols and Processes, Patients, People, Personal Protective Equipments (PPEs), Pharmaceuticals, Places, Preparedness, and Politics.

**Priority:**

to maintain cancer care as a key priority in the health system response even during a global infectious disease pandemic.

**Protocol and processes:**

to develop a set of Standard Operating Procedures (SOPs) and have relevant expertise to man the Disease Outbreak Response (DORS) Taskforce before an outbreak.

**Patients:**

to prioritize patient safety in the event of an outbreak and the need to reschedule cancer management plan, supported by tele-consultation and use of artificial intelligence technology.

**People:**

to have business continuity planning to support surge capacity.

**PPEs and Pharmaceuticals:**

to develop plan for stockpiles management, build local manufacturing capacity and disseminate information on proper use and reduce wastage.

**Places:**

to design and build cancer care facilities to cater for the need of triaging, infection control, isolation and segregation.

**Preparedness:**

to invest early on manpower building and technology innovations through multisectoral and international collaborations.

**Politics:**

to ensure leadership which bring trust, cohesion and solidarity for successful response to pandemic and mitigate negative impact on the healthcare system.

## Introduction

The COVID-19 pandemic has struck the world with whirlwind fury, afflicting over 172 million persons and causing over 3.7 million deaths by 7 June 2021 (World Health Organization, 2021b). Cancer care has been significantly altered with reduction in clinic consultations due to patient fears of coming to hospitals as well as deliberate postponement of visits to manage workload and to avoid inadvertent exposure of patients (Ranganathan et al., 2021). Treatment schedules have also been modified to deal with the crisis (Chiang et al., 2020; Kanesvaran et al., 2020). Cancer screening has been affected as a result of various restrictions and redirection of resources to manage the pandemic. This could have significant implications in the detection, management and clinical outcomes of cancer patients during and after the pandemic (Hanna et al., 2020; Basu et al., 2021; Ranganathan et al., 2021).

The devastating effects of COVID-19 seen worldwide have underscored the need for even better preparation for the next pandemic. This next pandemic has been called Disease X, a placeholder name for any future severe infectious disease that is still unknown to humans (Iserson, 2020). In fact, the likelihood of multiple future pandemics and their possible devastating impact in view of an increasingly interconnected world has already been recognized for some time (Heymann, 2006; Heymann et al., 2013). The threat is even larger if a more infectious and deadly virus strikes which has a longer and silent incubation time. The threat is significant, especially to patients with cancer, who have a greater risk of fatal disease if exposed to the infection and who also face a grave threat of long-term consequences in delay of cancer detection and treatment (Dai et al., 2020; Yang et al., 2020).

The best time to start preparing for the next pandemic is now and, to address these concerns, the Asian National Cancer Centre Alliance (ANCCA) (Gatellier et al., 2020) has put together the 9 “Ps” below as guidelines to help cancer programs around the world prepare for the next pandemic.

*Priority*

While there were 2,589,548 reported deaths due to COVID-19 in 2020, there were 9,894,402 deaths due to cancer during this same period (Hwang and Khoo, 2021). In fact, the magnitude of the ongoing ‘cancer pandemic’ has been profound both in terms of the number of patients succumbing to it every year as well as the consumption of financial resources and healthcare capacity due to the complexity of cancer care. The postponement of cancer care and screening as well as clinical trials and new developments due to the restrictions imposed from COVID-19 could result in a further increase in the global cancer mortality in the coming years. Thus, it is important to maintain cancer care as a priority in the health system response even during a global infectious disease pandemic, otherwise we could lose sight of the even more important cancer pandemic (Basu, 2020; Hwang and Khoo, 2021) and face an increased number of cancer casualties as well as considerable financial challenges linked to delayed cancer screening, treatment and care (Dewi et al., 2020; Gatellier et al., 2021). Consequently, national authorities should actively engage stakeholders to ensure that measures are taken to prepare for the next pandemic so that effects on patients with cancer are minimized (H.R.H. Princess Dina Mired, 2020; News, 2021).

Screening programs should continue during such a pandemic to prevent an upsurge in late-stage cancers subsequently. There should also be prioritization of therapy for curable cancers combined with close integration of supportive and palliative care in the management of patients with more advanced, metastatic cancers. Successful integration has been demonstrated to improve quality of life, quality of end-of-life care, better understanding of illness, physical and psychological symptoms, survival and patient’s satisfaction as well as to reduce futile oncology treatment, family burden, and cost of care (Hui et al., 2015; Vanbutsele et al., 2018; World Health Organization, 2021a).

*Protocols and Processes*

It is important to develop a set of Disease Outbreak Standard Operating Procedures (SOPs) for general patient management and cancer-specific patient management before the next pandemic happens and to appoint qualified and competent individuals with the relevant expertise to be in the Disease Outbreak Response (DORS) Taskforce, they should meet and review procedures regularly even before an outbreak. 

An example of the contents of a set of ready-to-go SOPs for the DORS manual is as follows:

*1. Disease Outbreak Response Condition (DORSCON) Framework*

This should incorporate disease classification into different levels of involvement with associated plans, for example, Green for conditions with negligible to low public health impact with no disruption; Yellow for those with low to moderate public health impact where cases may be present with imposition of some screening and border control; Orange for situations with moderate to high impact where cases or clusters are seen with imposition of quarantine, temperature screening and visitor restrictions in hospitals; and Red for those with high impact where there is widespread transmission with resultant school closures, and many other socioeconomic disruptions.

*2. Composition of DORS taskforce*

There should be separate ones for national, regional, and institutional levels where broader policies, specific issues, latest updates, and ground-level challenges are discussed. Individuals should be appointed to this DORS taskforce ahead of any crisis for fixed renewable terms and be trained for their respective roles. The role of the taskforce would be to address issues related to disease outbreak and formulate policies and guidelines to contain disease transmission as well as to ensure a safe environment for patients and healthcare workers. The taskforce leadership should comprise a Chair who would assume full responsibility for the taskforce and report directly to the Institute or Hospital director. There could also be a Deputy Chair as well as secretarial support headed by a main taskforce coordinator. There should also be Leaders in charge of Epidemiology, Surveillance, Laboratory testing, Contact Tracing, Case Management, Temperature Surveillance, Scheduling of Clinical Services, Risk assessment and Infection Control, Information Technology and Data Management, Materials Procurement and Management, Operations and Facilities Maintenance, Staff Welfare and Support as well as Crisis Communication. Their roles would be to provide input from their respective departments on issues arising during the pandemic and feasibility of various solutions proposed as well as to execute the needed tasks. There should be alternates members for all of the above leaders and a clear line of command.

*3. Considerations in planning should be based on lessons from the current pandemic and include the following*

a. Information technology and data management – including support with electronic medical records, data management, and novel equipment as well as the use of telemedicine to reduce patient need to come to the clinic and, if possible, remote access of hospital information systems for working from home. Setting up the necessary data management and logistics systems to allow for tracking of people movement, supplies and situation analysis.

b. Operations – including running and maintenance of the control room, hand hygiene, shower facilities, medical records, patient flow, staff flow, and staff dining facility.

c. Scheduling of clinical services – to haveready-to-roll modified schedules for chemo and radiotherapy to include infection control protocols to safely treat patients (patients infected or under surveillance) with radiotherapy if treatment delay is not desirable.

d. Materials procurement and management – to ensure sufficient and uninterrupted supply of supplies, especially of personal protective equipment (PPE), and optimize usage of available resources for greatest benefit.

e. Medications – ensuring adequate supply in case there are problems in shipping of medications due to border closures. Drive-through pharmacy to allow patients to collect medicine in a safer manner. 

f. Infection control and risk assessment – should not be confined to just the hospital or outpatient centres and should including measures to prevent nosocomial spread and concerted efforts to ensure up-to-date vaccinations for all the staff and patients.

g. Triage stations – which should include automated health declaration and temperature tracking.

h. Healthcare worker (HCW) temperature surveillance – to ensure automated tracking, follow up actions and enforcing compliance, including non-clinical ones in the backend offices.

i. Contact tracing, epidemiological surveillance and monitoring in event of any exposed staff or patients, both at work and at home.

j. Staff welfare and support – which should include their treatment if they get infected as well as food supply and temporary accommodation arrangement for staff in the event of an outbreak.

k. Crisis communications – these should include internal and external updates in risk and the crisis situation.

*4. An organization or functions chart would be helpful to have clear reporting lines and interaction of various elements in the taskforce.*

All members of the DORS taskforce should be part of the activation and call tree to be able to respond immediately in an emergency. Such a call tree should be exercised regularly in peacetime.

*5. Risk assessment and updates from international and local data by an infectious disease expert.*

It is important to remember that new pandemic is in fact an unknown pandemic and the cancer care community should have a contingency plan of preparedness addressing the emerging facts of a progressive pandemic.

*6. Develop a risk stratification grid and defined responses depending on community positivity rates for the disease.*

*Patients*

Clinical safety of patients should be of utmost importance and all measures taken, even if cancer follow-up is affected, this factor should be taken into consideration.. Clear segregation of hospital clinical areas is necessary between patients infected by the pandemic and those visiting for non-pandemic care. Patients requiring palliative care should limit their follow up in hospital, therefore integrated services in the primary health care to include home care is important. Telemedicine, including virtual consults and virtual tumor boards meetings help to ensure continuity of care and updating of treatment plans for better clinical outcomes while decentralizing care for patient protection. Electronic payments, medication delivery and decentralized treatment at day centers or patient homes have been implemented in several cancer centers to reduce patient dwell time in the cancer center. 

It is also important to obtain regular feedback from patients about their concerns and suggestions for improvement and to build awareness programs to help educate patients on how to look for early signs of infection. Patient hotlines can be set up using a few options of commonly used media channels which can be readily accessible by the patients and families. Cancer control could also leverage on the use of big data and artificial intelligence to optimize the process of early detection and screening of early-stage cancers. Therefore, the new normal health care should be implemented to optimize the delivery of cancer services.

*People*

Human capacity of healthcare systems will be strained and put to test in dealing with emerging disease outbreak (Lum et al., 2020; Ong et al., 2021). Surge capacity and business continuity planning should include collaboration and partnership with other healthcare providers in the country and from outside the country where feasible. Such planning should consider of the following:

1. Sufficient buffer manpower needs to be available that can be called upon in event of an outbreak with policies for staff rotation.

2. Staff need to be regularly trained to know what to do in event of an outbreak, including training for multi-tasking situations.

3. Plans for split teams if needed with staggered working schedules.

4. Programs to ensure psychological and welfare support of staff during outbreak.

*Personal Protective Equipment (PPE)*

From the COVID-19 crisis, we have witnessed PPE shortages in the healthcare systems of many countries during the start of the pandemic. Therefore, it is important for the healthcare systems to have adequate buffer stocks for PPE, which include national stockpiles. Importantly, supplies should be prioritized for healthcare workers and frontliners. Furthermore, there should be clear guidance regarding types of PPE that are appropriate even for healthcare workers depending on the clinical area in the hospital that they work in. 

Each country should also build up capacity to manufacture own PPE and companies should have plans to switch manufacturing to support the health sector if needed. To ensure proper usage of PPEs and prevent hoarding, wastage or bidding price wars for PPEs, a national stockpile system should be complemented by education for the public on proper usage and disposal of PPEs as well as inventory tracking system to monitor and predict the demand of PPEs and adopt measures to ensure continuous supply of PPEs in different healthcare facilities (Ma and Tsai, 2020; Chiang et al., 2021). Additionally, local or national guidelines should be developed and disseminated in an event of the need to re-use of PPE under specific situations, in order to mitigate risk and improve the safety of staff and patients.

*Pharmaceuticals*

During the COVID-19 pandemic, a panoply of recommended treatments appeared over the internet. These included the use of hydroxychloroquine, convalescent plasma, anti-IL6 antibodies and many others. In fact, many of these treatments were attempted in desperate situations, but with some controversy about the results. There were many widespread claims on social media from individuals or groups on unproven treatment for the infection as well as putting fear or hesitancy into the public on vaccinations. This calls for a more concerted way by the national and international experts and authorities to address such misinformation. As cancer patients are a particularly vulnerable population, with several times increased risk of death if infected with COVID-19 during cancer treatment, they need to be placed in high priority to receive proven treatments. However, given the many unknowns in a new pandemic, definitive proof may be lacking, and strong international efforts should be taken to carry out good collaborative clinical trials, particularly in this vulnerable patient population. There should be a central team, which can analyze the evidence for the efficacy of available and proposed treatments with dissemination of the evidence and recommendations as well as stringent measures to discourage the use of unproven therapies. Unified, evidence-based guidelines are essential to avoid confusion amongst treating physicians and patients.

The unprecedented speed of development of COVID-19 vaccination has brought new hope that this crisis could come to an end. In many countries, health care professionals (especially those in the frontline) and vulnerable populations like chronic patients and the elderly were given first priority for vaccination. However, there are wide disparities in access to vaccines for patients with cancer, not only in low- and middle-income countries, but also in high income countries (Yusuf et al., 2021). Cancer patients, who have no contraindications, should be given top priority for vaccination given the vulnerability of this population and similar prioritization should be given to their caregivers and healthcare providers. However, those on active immunosuppressive treatment could have blunted responses to the vaccination and some initial vaccination programs have excluded this group.

Many member states have reported challenges in ensuring adequate supply of essential medicines and pharmaceutical products for cancer treatment, especially among states which do not have adequate manufacturing capacities. Effective regional and international collaborations are required to ensure supplies and delivery of essential products such as chemotherapy drugs. Capacity building in technical expertise to manufacture essential products should be part of national development planning for member states. 

ANCCA could facilitate the formation of a regional committee or body to help member states with less resources or technical capabilities so that every country is prepared and equipped with the basic know-hows in manufacturing the essential medicines and PPEs to sustain through the pandemic when the supplies run low especially when there are borders closure and delays in import and exports.

*Place*

During a pandemic, facilities will need to be built or reconfigure to cater for infection control triage and screening sites, so hospitals should be designed to cater for potential future reconfigurations. Importantly, hospitals and cancer centers should provide clear instructions on separate patient pathways and staff flow to minimize risk of transmissions of infection. There should be demarcation of places which can be used flexibly for the isolation or regular cancer care as per the need. Strict measures taken to include catering for sufficient isolation rooms or being able to build or designate new ones very quickly, having segregated areas for different categories of patient and staff, as well as being able to modify dining areas and office areas to minimize the risk of infectious disease spread. New building should also have infrastructure designed to cater for potential future large outbreaks. Having dedicated infectious disease hospitals would be an advantage as well as drawer plans to convert large convention venues and other locations into community care facilities if needed (Chia et al., 2021). Member states with segregated care model of patients with cancer, for example Brunei and Singapore, have allowed for the continuation of cancer care even when there were outbreaks in the community (National University Cancer Institute of Singapore Workflow, 2020). Such models include hospital dedicated for cancer patients only as well as segregation of departments within a hospital such that there is no mixing of patients to reduce the risk of cross contamination. Procedures to disinfect potentially exposed or contaminated places should be in place and exercise during peacetime to ensure compliance and adequate decontamination. Integration of palliative care in oncology to reduce the length of hospital stay is applied by providing continuing care at patient’s home or domicile. for terminally ill patients, for whom all treatments have been exhausted and progression cannot be halted, a more comfortable and safer place at home is required to avoid emergency room admission and hospital death.

*Preparedness*

The success of preparation for the next pandemic goes beyond what any cancer center or hospital can do. The COVID-19 pandemic has led to new global initiatives toward positive changes for better cancer care (Lombe et al., 2021). National policies and plans should be in place and should extend beyond the Ministry of Health to involve all other Ministries, especially that of Immigration, Transport, Finance and Civil Defense. 

International collaborations undertaken by NGOs like that of the ANCCA would be important to allow timely sharing of technical information, measures taken and lessons learned between cancer centers, as well as avoiding delay due to bureaucracy of government channels. International collaborative seminars could be organised regionally to raise awareness on lessons learned in managing oncology patients during the pandemic. Dissemination of timely and accurate information through online seminars should be organised regularly for the staff, patients and public. ANCCA could also facilitate collaborations among cancer centers in the clinical trials of cancer related treatment for earlier approval and manufacturing in the region. Financial sustainability will pose a problem in the aftermath of any pandemic and cancer centers should develop buffer resources to tide through the crisis. Health programme will need to be restructured to ensure minimal disruption to cancer care while remaining cost effective. Early investment of capacity building in technological innovations, such as virtual consults and artificial intelligence, could enhance patient care while managing scarce manpower resources in a crisis. Following this publication, ANCCA intends to conduct a survey of the preparedness index among Asian national cancer centres for the next pandemic based on the various categories discussed in this paper.

*Politics*

Leadership in the implementation of effective strategies as well as sustainable financing are necessary for a successful response to a future pandemic. Interestingly, in a 23-nation comparative study of COVID-19 responses, it was found that the politics of implementing healthcare interventions rather than the actual policies themselves, were most important in determining the outcomes (Su et al, 2021). Importantly, trust, cohesion and solidarity in each country were crucial in ensuring smooth implementation of national measures, regardless of whether the country was more authoritarian or democratic, as long as there was a good degree of trust and idealism (Jasanoff, 2021). As such, while cancer center and health ministries can do a lot to prepare for a crisis, governments should strive towards building robust pre-emptive strategies and strong socio-economic foundations to ensure resilience in event of future catastrophes (Dzau and Balatbat, 2020).

In conclusion, while the COVID-19 pandemic has caused various degrees of disruptions in many healthcare systems including cancer control, we should evaluate the lessons learnt, leverage on the silver linings arising from the crisis and re-examine our strategies, processes, and guidelines on cancer control. Efforts on cancer prevention and early detection need to be increased to ensure prompt diagnosis and management of cancer and lessen the burden on the healthcare systems when another global health crisis emerge in the future. In addition, practical outcomes and recommendations from current and upcoming research related to the impact of COVID-19 on the increase in cancer deaths as well as the escalation in healthcare cost from delayed diagnosis of cancer and late stage cancer treatments are also urgently needed. This “9 Ps” guide put together by ANCCA aims to serve as a summary guide or reminders for cancer centres or programme to better prepare for the next pandemic.

## Author Contribution Statement

William Hwang and Laureline Gatellier contributed to this work as the last and first authors respectively and were responsible for the conception and design of the manuscript; literature review, drafting, revising, formatting and approval of the manuscript. Sokking Ong contributed as the second author and were responsible for the literature review, drafting, revising, formatting, approval and submission of the manuscript. Tomohiro Matsuda, Noraslinah Ramlee, Fennee Lau, Suhana Yusak, Soeko Werdi Nindito D, Achmad Mulawarman Jayusman, Ayu Hutami, Kishore K. Pradhananga, Aasim Yusuf, Giang Nguyen Huong, Alireza Mosavi Jarrahi, Tatsuya Suzuki as co-authors from the various national cancer centres in the Asia region were responsible for the literature review, revising, editing and approval of the manuscript.
